# The Nursing Supplement

**Published:** 1888-08-18

**Authors:** 


### The Nursing Mirror.

All Contributions for this Supplement should be addressed "The Nursing Mirror," care of The Editor, 38, Old Jewry, E.C.

### En Passant.

IRISH ECONOMY.—*Sister.*—"Nurse Bridget, why do you break that chunk of ice in two pieces before putting it into the box?" *Nurse Bridget*—"Faith, Sister, it lasht the longer. Two paces will lasht longer than wan; they kape aich other cowld."

LONDON SCHOOL OF MEDICINE FOR WOMEN.—The names of the following students of the London School of Medicine for Women appeared in the pass list of the Intermediate Examination in Medicine of the University of London issued on Tuesday: Miss Berthon, 1st division; Miss Dove, 2nd division; Miss Tribe, 2nd division; Miss Staley and Miss Pace, excluding physiology. In the honours list. Anatomy. Miss Longbottom, 1st class, ; Miss Sturge, 2nd class; Miss Benson, 3rd class. Physiology and Histology. Miss Benson, 2nd class; Miss M'Laren, Miss Madgshon, and Miss Williams, 3rd class. Materia Medica and Pharmaceutical Chemistry. Miss Benson, 1st class; Miss Farrer, 2nd class; Miss M'Laren, 3rd class.

ELIZABETH FRY.—That Mrs. Fry was a forerunner of Florence Nightingale, and the first to introduce a system of nursing in England, is a fact almost forgotten. Yet it was in 1840 after a visit to Pastor Fliedner at Kaiserwerth, that Mrs. Fry, with the assistance of her sister Mrs. Samuel Gurney, established the first home for nursing sisters in the city. The nurses wore a plain unconspicuous dress, and were paid by the institution and forbidden to receive presents. To the poor their services were gratuitous, but their aid was highly valued by persons of all classes, including royalty.

FLORENCE NIGHTINGALE.—As every word written by Miss Nightingale is eagerly received by our readers, we publish the following from a letter written to a lady at Helensburgh with regard to the recent death of Miss Beatrice Clugston: "We ought to bless God for such a life as dear Miss Clugston's—such a work. And now what a glorious rest is hers! Who can wish her back? But her works will live after her. Indeed I feel the deepest gratitude to you for writing to me; it was very, very kind. And what a comfort to you to know what a blessing you were to her life! Excuse a brief note; my thoughts about her are not brief. But all this year I have been seriously ill, yet always under the severe pressure of work when work I could. God bless you and her great institutions.—Ever sincerely yours (signed), FLORENCE NIGHTINGALE."

MEDICAL WOMEN.—At the July sittings of the examiners of the Conjoint Board of the Royal Colleges of Physicians and Surgeons of Glasgow, the following lady candidates passed the respective examinations: *First Examination*—Lillian V. Cooper, Luton, Kent; Elizabeth M. Harris, Calne, Wilts; Charlotte M. Wheeler, Crewe. *Second Examination*—Jane B. Henderson, Bo'ness; Agatha Porter, Glasgow; Louisa R. Cooke, Yorkshire; Mary L. Gordon, Liverpool; Catherine M. Wickham, Dublin; Lillias Hamilton, Australia. *Final*—Janet Hunter, Ayr; Sarah Grey, London. We believe most of these were students of the London School of Medicine for Women. Miss Waterson, M.D., formerly a student of the same schoool, has just passed the examinations of the Psychological Society for a certificate for mental diseases, and is the first lady who has done so. Women are certainly beginninng to do well at examinations, 126 ladies have taken the L.L.A. degree of St. Andrews this year.

A NECESSARY VIRTUE.—"A nurse should be truthful, not only speaking the truth, the whole truth, and nothing but the truth, but capable of observing truthfully, and of reporting correctly. To do this, all the special senses must receive a special training—the eye, the ear, and the touch must become so sensitive and accurate that they will be quick to appreciate all the varying changes which shall occur in the sick, with a memory so retentive as to report truthfully all they have observed. This training of the special senses, so that their observations shall be of value, is no easy task; months and years of patient labour are required. It is this more perfect training of the senses that makes one physician surpetior to another, and one nurse more reliable than another."—*Professor Loomis, M.D.*

THE POLICE COURTS.—Those who ever glance through the police news must be surprised to notice how often there appear references to nurses in the evidence given. Generally it is some poor old pauper full of troubles and complaints who comes to have his grumble out before the magistrates. Of course he has several grievances against the nurses as well as against the other workhouse officials. We ourselves have grumbled at specimens of union nurses ere this. An inmate of the Lewisham Infirmary made a remark about a nurse she did not like, whereupon the nurse fetched a porter, who used extreme measures with the grumbler, who now accuses him of assault. But, you see there was a woman at the bottom of it, as usual. Then there was a case of a surgeon's wife who knocked a lady about with an umbrella, under the belief that she was a nurse of whom the foolish wife was jealous. Evidently, a nurse is not always superior to the common failings of her sex.

AN IRISH ISLAND.—Sir Maurice Fitzgerald has been writing to the *Morning Post* about a case of religious intolerance at Valentia Island. A new nurse was required for the village hospital, and the selected candidate was a Protestant. The parish priest, according to Sir Maurice, gave out that he would do all in his power to prevent his parishioners coming under her care, and should they do so, he would refuse them the last sacrament and curse them from the altar. Now this story is one of such bigotry that we fancy there must be another side to it, and that the language of the priest has been exaggerated, or there was some untold reason for its force. Sir Maurice Fitzgerald is Knight of Kerry, and owns the whole of Valentia Island. Like many other Irish landlords, he has not got all his rents lately, and perhaps his spirit is embittered. From personal experience we know the kind heart of the priest, who is a friend of the Bishop of Kerry, and we know how patient and hard-working are the dwellers on Valentia! Yet we in England are called "insular" by our Continental neighbours, and Ireland is an island off England, and Valentia is an island off Ireland. No wonder narrowness flourishes in such a place quite as much amongst the Protestants as the Catholics; and the fact that Sir Maurice no longer rules so absolutely over this small piece of land is only proof that the island is not quite so be-knighted as of yore. We quote the following few lines from the Knight of Kerry's letter, at the same time deprecating its tone: "Although the present hospital was erected with funds supplied by Protestants in the neighbourhood and elsewhere, the late nurse was a Roman Catholic, and I have thoroughly satisfied myself that the religion of Sister F. did not in the smallest degree influence the committee, and that the election was made solely and entirely because the committee was convinced that the appointment was the best that could be made. Three Roman Catholic assistant nurses have been compelled by the personal interference of the priest to leave the hospital." It is always a pity when religion and charity are at variance, especially in regard to nursing.

### Original Articles.

### A NURSE'S PROGRESS.—FROM APPLICATION TO CERTIFICATION.

### Paper II.—Probation.

With what an anxious heart the new probationer steps out of the cab when it draws up before the nursing home ! Luckily the Home Sister is ready to receive her and show her her room, and offer the usual friendly cup of tea. It is always better to arrive at a hospital or a house about four o'clock, for shyness is generally conquered at the tea-table, and friendships are more easily then commenced. Of course the new probationer comes down to the Home Sister's room with her cap wrongly put on, and several small faults visible in her uniform. All this the sister puts right, and then, tea over, she leads the probationer along dim stone passages, up and down stairs, and finally into a long light ward where some thirty women are lying in bed. Here she introduces her to the sister of the ward, who in her turn passes the probationer on to the nurse who is busy making the beds. The next three hours till the supper-bell rings are the hardest in a nurse's life. She is too embarrassed to be of much use, her hand shakes with nervousness, and her face burns with blushes. The nurse is wearied by her long day's work, and too anxious to put all straight for the night before nine o'clock comes, to spend much time with her probationer. The patients are rather amused at the appearance of such a woefully-shy and ignorant person in a cap and apron. Those who are pretty well condescendingly teach this young nurse what to do, and how to do it; but the old women are querulous and rude under the touch of strange, untutored hands. At last the night nurses come in at the door, the bell rings, and the nurse comes forward to show the probationer the way to the dining-hall. It is a dreadful ordeal to sit down at those long tables amongst the crowds of women all dressed alike: but the probationer soon discovers that the others are too tired and hungry to pay much attention to her, and, being too excited to eat herself, she sits and watches the faces of those around. After supper comes prayers, and then bed. The ice has been broken, the first plunge taken—our candidate is at length a probationer-nurse.

The first experiences of nursing life are strange and trying. The ravings of delirious patients, the moans of the restless, the crying of the children, all seem unbearable at first. Then the probationer has often to make her first close acquaintance with death. Unless she is thoroughly in earnest in her work, these first experiences will be too much for her, and she will be beaten from the field. Not long since a probationer left a large hospital because she found out that the patients sometimes died, and she was afraid of dead people. It is no use to slur over the disagreeables of hospital life, but it can be confidently pointed out to all candidates that a good woman can always rise superior to these disagreeables, and perform the most menial tasks with gentleness and nobility. There are a great many merely domestic duties demanded of a probationer, and instead of being disgusted with them she will do well to give them her chief attention at first, until she becomes used to hospital sounds and scenes. As the actress begins her career as a "walking lady," so the nurse begins hers as a maid-of-all-work; and stage-fright can hardly be more necessary to conquer before taking a prominent part than hospital-fright, which may for ever mar a nurse's character with the doctors on whom she attends. To polish brass and peel potatoes is not soul-stirring labour, but it is good discipline, and the woman who is disagreeable over small things will never be pleasant over great. During these first days the probationer suffers much from sore feet and aching back, the result of an unusual amount of standing. The cruelty displayed by some sisters, who never like to see a probationer sitting down, is simply extraordinary. There has been a great deal of talk lately about "fagging" amongst nurses, but the remedy is in the hands of the sisters. If they would only try to remember how strange and tiring hospital life was to them at first, they would surely be more thoughtful of new probationers. Great care of health is necessary during the first few weeks in a hospital, and there is no excuse for overworking a probationer or disregarding her evident weariness. "I had a hard time myself, and I always give my probationers as much hard work as possible," said a sister lately. This is a spirit which ought not to exist.

The etiquette of hospital life is very strict, and non-observance of it will often bring a nurse into shame or trouble. With regard to the patients, it chiefly consists in not talking too long to them, especially not showing any familiarity with them, nor any preference for one more than another. With regard to the sister, it consists in always standing in her presence and addressing her respectfully, no matter whether the probationer or sister is of the higher social standing. These things do not enter into hospital life. As the head of the ward, the sister must always be treated with regard and listened to with attention. To lean against a table or put your hands in your apron pockets while the sister is speaking to you are signs of a want of natural politeness. These small details must be attended to if order is to be maintained in a large establishment, and the probationer who feels humiliated by rising when the sister approaches her has a false pride which unfits her for hospital life. The new probationer comes very little into contact with the doctors or medical students; indeed etiquette consists rather in avoiding them. It may seem strange that a fact noticed by the probationer with regard to a patient must be told the nurse, who will tell the sister, who will tell the house-surgeon, who will tell the visiting surgeon, but so it is. The outside world is only too ready to sneer at the nurse and the student, and to hint at flirtations. Nothing is more degrading and humiliating to nurses as a body than to get some foolish girl into their ranks who will so disgrace them. Hence the barriers built up to prevent the spread of scandal and to maintain order and discipline. When in doubt how to address a physician or student whose name is unknown, there is no reason why a nurse should dislike saying "Sir." It is a very simple title, and a probationer's dignity is more injured by hesitation in using it than in frankly speaking it. "I always used to say 'Sir.' Then they didn't think I was a lady pupil, and left me alone," was the remark of a clever nurse. The "lady pupil" is only too apt to be the butt of the students' witticisms if she shows any signs of her supposed superiority to her fellow-nurses.

One common fault amongst new probationers is too eager a desire to learn. In season and out of season they pester the nurse to tell them the meaning of this or that word, or bother the sister to explain to them some course of treatment. The general result of such thirst for knowledge is a severe snubbing, and a recommendation to learn how to dust and clean knives properly before attempting higher things. Yet it is a most laudable desire, this wish to understand all that is going on around; and if only a little tact and patience is shown, the probationer can generally achieve her wish. Advantage must be taken of such quiet minutes as there may be to ask questions, for when a patient is in a fit, or the morning sweeping has just begun, it is only natural that ignorant queries should be sharply answered. A great deal can be learnt by watching and waiting, without irritation to others; and, while never losing a chance of seeing a dressing done or a remedy applied, the use of eyes and intellect often teaches more than the use of the tongue. A probationer is usually put in a medical ward first, and she should, before she leaves it, have learnt how to use the clinical thermometer, how to give enemata, to make beds and wash helpless patients, and the means of preventing bed-sores. These are merely a few of the simpler things a nurse may well demand to see and understand in the course of a month or two. It seems absurd, yet probationers have often been known to spend six weeks in a medical ward without ever learning how to chart a temperature. Of course, the fault in such cases must lie on both sides—on the side of the sister, one of whose chief duties is to train nurses, and on the side of the probationer, who either had no desire to learn, or was clumsy in her mode of showing it.

### Everybody's Opinion.

[*Correspondence on all subjects is invited. No communications can be entertained if the name and address of the correspondent is not given, or unless one side of the paper only be written on.*]

### PRIVATE NURSING.

SATISFIED AND APPRECIATIVE.

I HAVE been a constant reader of THE HOSPITAL since its first publication. It is very kind of you to allow us to express our opinion so freely. I have often wished to say a few words, but my want of education kept me silent; but now, with your permission, I will say a word or two about private nursing, and as I am one of the very old nurses, trained twenty years ago, I know you will not expect much from me, but will kindly excuse all my defects. I fancy I can hear some of the highly-trained nurses of the present day exclaim: What can she know about nursing? But as I have had twelve years' experience of private nursing, I think I do, or ought to know, a little about it. I do not think it is at all the hard life that "Islands," would lead one to suppose from her letter. I like the work, and find it very pleasant. It is anxious and a little hard when the patient is very ill, but that does not last long, and then he is convalescent, when the nurse has little or nothing to do. She can therefore read, work for herself, or do any thing else she likes, when she is not in attendance on the patient. Then I get well paid for my services, and in nineteen cases out of twenty the patient and his friends are most grateful to me. Now and then I do meet a patient on whom it is not a real pleasure to attend, but those are very few, and then I say to myself, Never mind; he will be better soon, and then I can get away. On the whole, I think if a nurse is fairly strong and will accommodate herself to circumstances, her life is a very pleasant one, for she gets some very nice changes and she meets with some very kind people. Perhaps "Islands' was not well when she wrote to you, or, worse still, she may have the misfortune to belong to one of those institutions where the nurses are treated not like women, but as school-children. I consider myself fortunate in belonging to one where we are liberally and kindly treated, and when we are not at work we do what we like and go where we like. I thank you heartily, sir, for taking such an interest in us. I feel sure every nurse must feel very grateful to you.

AN OLD NURSE.

DISSATISFIED AND ABUSIVE.

MAY I beg leave to add a few remarks of my own to the sensible letter of "Islands" which appeared July 21st? The lives, not of private nurses alone, but of nurses in general, are made more dull and dreary than they need be by petty restrictions and interferences with personal liberty, which would not be endured for a week by any class of working men. Regulations are often made, as frivolous as vexatious, which are never dreamed of by the general public who support hospitals, &c., and if they were would be speedily swept away by the indignant scorn of healthy public opinion. A nurse's life is at best a very hard one, and in, I fear, the majority of cases, the principal wish of her superior is to take away every chance of its being casually softened. Not long ago I was told that at a large provincial hospital the nurses were not allowed to sit down after fourteen hours' hard work, if they had a few minutes to spare before going off duty. Think of that, kind folks, who clamour for seats for shop girls. Then again many institutions require their nurses to wear out-door uniform when off duty, which practically debars them from every recreation where such a costume is unsuitable. The style most frequently chosen is ludicrously unsuited to either work or pleasure. Why should a nurse be made to look like a member of a religious order, which she probably has never been, or a widow "open to offers," which she has no desire to be? It is a lesson in cynicism to note how often ladies of society, who permit themselves every extravagance of dress or amusement, will be eager to deprive nurses of such means of enjoyment as the little time and money usually at their command may allow them to procure. The scriptural injunction most usually followed seems to be, "From him that hath not shall be taken away even that which he hath," rather than that which says, "All things that ye would that men should do unto you, do ye even so to them." All other classes are supposed to need times of social relaxation, when it would be "bad form" to remind them of their particular "shops;" but the nurse, who is expected to always have a supply of moral, mental, and physical strength to meet the needs of others' weakness, is never to have the bow quite unstrung. Out doors or in, she is always "nurse," till she probably learns to dislike the name she was proud to assume as the badge of loyal loving service to suffering humanity. A woman of spirit and culture who has, perhaps, left a position of her own in society for honest hospital work, does not feel flattered by the patronising notice bestowed on "Nurse" Smith by some dashing member of the plutocracy, who would be highly indignant if addressed as "Soap-boiler de Tomkyns."

I have often thought that nurses were expected to realise an ascetic ideal of life on behalf of those who had no wish to do so for themselves. Whether such vicarious sacrifices are beneficial to those who exact them or to those who have to make them I leave to psychologists to decide.

ANOTHER OLD NURSE.

### ZENANA MEDICAL COLLEGE, 58, ST. GEORGE'S ROAD, S.W.

THE Nursing Supplement of THE HOSPITAL for July 14th contains the following, in reference to which I would offer an explanation: "Young medical women who are intending to go to India, China, or Africa obtain at the New Hospital training they can get nowhere else." What! I thought, after eight years of successful work is the Zenana Medical College, 58, St. George's Road, S.W., of which I enclose a report, still unknown? Surely there must be some mistake, for numbers of ladies have been trained there, and are doing valuable medical mission work in India, China, Syria, and other eastern countries. The curriculum is for two years, during which time the students gain an immense amount of knowledge, as also of practical medical work, and after passing examination a diploma is granted by the society, every student having previously obtained the midwifery diploma from the Obstetrical Society. The maternity department is now in such working order that the students have abundance of cases (almost more than they can attend) besides courses of lectures, their services being much sought for by the poor of the neighbourhood. Closely connected with the College is the Hospital for Women and Children, 9 and 32, Lupus Street, S.W., where the students see patients and learn dispensing. Before me lies a communication from a student, who twelve months ago passed her examination with honours, having also obtained the midwifery diploma and a prize for dentistry. She has just been accepted by the Church of England Young Men's Society for medical mission work in Srinagar, and will (D.V.) sail for India in October. I might multiply similar cases, and tell of our students who are now abroad in charge of hospitals and dispensaries, as well as visiting in the zenanas as medical missionaries. The work at the college is only limited by want of funds. Just now we are seeking to form free scholarships for two Syrian girls—pupils of one of our former students, who feels the immense value of her own training in her present sphere of mission work. For this purpose we need two hundred guineas, which would give them residence, board, and instruction for two years. Shall we plead in vain? Dr. G. de G. Griffith, the hon. sec., will gladly give all information concerning the work, and friends are invited to visit the College, and so become personally acquainted with the details. Having been in India, I can bear personal testimony to the value of this work. More especially as during a long and serious illness in Amritsa I was attended by ladies trained at the college. I feel sure that all who read the account of work done during the past year will be deeply interested in it. I commend it and the institution of which it speaks, to your earnest attention and that of your readers, and remain

M. A. ROBINSON, Zenana Missionary.

### Miss John's Nurses' Home at 1, Kerrsland Street, Glasgow.

THE popular charge that private nursing institutions deserve some of the obloquy that has fallen on the "sweating system" cannot be justly applied to the Nurses' Home at 1, Kerrsland Street, Hillhead, Glasgow, where Miss E. Johns is sister-in-charge. There the nurses receive the whole of their earnings, with the exception of a small weekly sum to keep their room in the Home while they are out at cases. For the benefit of the public, it may be stated that the terms charged for the nurses are moderate, though of course sufficient to satisfy the legitimate demands of a trained worker. The regulations for the treatment of the nurse given to the friends of the patient when she is engaged are reasonable, and very necessary for the enlightenment of those people who think a nurse is a machine of marvellous efficiency, not a human being as likely to feel the effects of work and worry as themselves, and who therefore make demands on her strength which "a hospital-trained angel with a cast-iron back" could not stand. Of course these *exigeant* folks are in the common phrase "fools for themselves," and act in ignorance of the common-sense dictum which heads these regulations: "To overtax the physical powers of a nurse is to risk her efficiency, and must necessarily lead to some relaxation of vigilance and attention to the patient." One rule is that "the nurse should not take her meals either in the sick room, except in cases of necessity, or with the servants." Surely it is time that such an injunction should not be needed, and that people should learn to behave to the brave and educated women who come to their help in times of sickness with the consideration and respect their position deserves.

### Notes and Queries.

TO CORRESPONDENTS.—1. Questions may be written on post-cards. 2. Advertisements in disguise are inadmissible. 3. In answering a query please quote the number. 4. If a private answer is desired, a stamped addressed envelope must be forwarded.

(45) *Training in Midwifery.—Service in Lieu of Fees.*—Nurse Brown writes from 107, Twycross Street, Leicester: Can you tell me of any lying-in institution where, instead of payment, they will take me as a pupil in midwifery, and retain me as a nurse at a small salary for one or two years?

(44) Nurse A. would like to know if there is any special house in London which makes nurses' uniforms, viz., dresses, cloaks, and bonnets, and also where the turn-down collars may be bought.

### Answers.

*Free Training in Midwifery.*—A. M. is informed that there is no place where free training in midwifery can be obtained. The usual fee is £26 5*s.* for three months. If A. M. does not feel able to undertake night-work, she is not fitted for the calling of a midwife, who is exposed to night-work in all weathers at all seasons. Her best course would be to train as a monthly nurse, at Queen Charlotte's or some other of the London lying-in hospitals. The inclusive fee is £11 0*s.* 6*d.*, less than half the cost of a midwifery training, while the chances of a successful career are greater, as it is very difficult to get into profitable practice as a midwife.

### Appointments.

[It is requested that successful candidates will send a copy of their applications and testimonials, with date of election, to THE EDITOR, The Lodge, Porchester Square, W.]

GATESHEAD CHILDREN'S HOSPITAL.—At a meeting of the committee held on the 3rd inst., Miss Annie Harper, of the Home for Trained Nurses, Bath, was elected matron of the above hospital, and will commence her duties on October 1st.

RAMSGATE INFIRMARY.—Mrs. Sarah Homer has been appointed matron of the Ramsgate Seaman's Infirmary and General Hospital.

MIDDLESBOROUGH FEVER HOSPITAL.—Miss Going has been appoinsed matron of this hospital, in place of Miss Proctor, who has gone to the Hull Sanatorium. Miss Going has been matro n of the Carlisle Fever Hospital for some years.

### The Nurses' Bookshelf.

[Boo ks for Review should be sent to The Editor, The Lodge, Porchester Square, W.]

### Home Nursing.*

THIS small manual is much on the lines of many other books of this sort, and contains little that is new. It is published at the request of the Health Society, and of many who have attended the author's lectures on nursing. To the last, who have had the advantage of seeing its precepts partly demonstrated, it may prove of use by keeping their memory fresh on minor points. Home nursing is a very difficult subject on which to write, as an ounce of practice is always worth pounds of precept. You might read pages on the making of the bed of a helpless patient, and not be much the wiser; whereas, once see the thing done, and all is easily understood. The delivery of these lectures, with demonstrations, has done much good in diffusing useful knowledge; and those who have heard the lectures cannot do better than buy the book, and so remember them.

### Diet for the Sick.†

WE are glad to welcome the third edition of this useful little book, especially since it contains some new recipes for nitrogenous preparations not derived from the flesh of animals. The method of making beef-tea given is far more thorough than that usually employed, but also more troublesome. The author, however, explains the reason of each stage of the process to encourage the nurse to carry it out properly and intelligently. The recipes for meat biscuits and for orgeat will be especially welcome to many. This small book contains twenty-five recipes for meal diets, twenty-eight for milk, twenty for whey, seven for digested foods, and seven for enemata. The nurse who is provided with this total of eighty-six recipes ought to be able to tempt the most capricious appetite, and to persuade the weakest patient to take sufficient nutriment.

### SEASIDE CONVALESCENT HOMES.

WE find that Mrs. Marsham's Convalescent Home at Brighton (referred to last week, page lxxv) is for women only. Miss Campbell should therefore apply to Miss Gould, lady superintendent, Miss Marsh's Home, Blackrock, Brighton. Non-subscribers pay 10*s.* per week. We are indebted to Mr. Henry N. Custance for this suggestion, for which we offer him grateful thanks.

### Vacancies.

The following vacancies are announced:—

### Matrons.

Carlisle Fever Hospital; £50.

Lytham Cottage Hospital.

City of Liverpool Infectious Diseases Hospital; £70.

### Nurses.

Crieff, Perthshire; £52 per annum, with increase.

Croydon General Hospital, night; £20, rising to £24.

Cancer Hospital, Brompton; several; £25, rising to £30.

Royal National Hospital for Consumption, Ventnor, I.W.

West Kent General Hospital, Maidstone; £25.

General Infirmary, Hertford; £25.

Nurses' Institute, Canterbury; several

### Probationers.

General Infirmary, Hertford; £12.
Nurses' Institute, Canterbury.

---

* By E. M. Homersham. National Health Society, 44, Berners Street.

† By Dr. James J. Ridge. J. and A. Churchill. Price 1*s.* 6*d.*

